# Intracranial Metastasis of a Primary Mediastinal Seminoma Mimicking a Convexity Meningioma: A Case Report

**DOI:** 10.7759/cureus.105759

**Published:** 2026-03-24

**Authors:** Rikiya Kameno, Shinjitsu Nishimura, Asuhito Takemura, Sumito Okuyama, Keiichi Kubota, Junko Matsuyama, Tadao Matsushima, Hideo Sakuma, Yuya Yoshida, Sadayoshi Watanabe

**Affiliations:** 1 Neurosurgery, Southern Tohoku General Hospital, Iwanuma, JPN; 2 Pathology, Southern Tohoku Research Institute for Neuroscience, Koriyama, JPN; 3 Medical Oncology, Tohoku University Hospital, Sendai, JPN; 4 Neurosurgery, Southern Tohoku Research Institute for Neuroscience, Koriyama, JPN

**Keywords:** convexity lesion, differential diagnosis, dural invasion, germ cell tumor, meningioma mimic

## Abstract

Convexity germ cell tumors are exceedingly rare and may closely mimic meningiomas, particularly in young men. This case report presents a rare convexity germ cell tumor characterized by dural invasion and skull destruction. An 18-year-old man presented with headache and nausea. Neuroimaging demonstrated a right frontoparietal extra-axial mass accompanied by adjacent skull hyperostosis and significant cerebral edema. Based on these radiological findings, a convexity meningioma was strongly suspected, and a craniotomy was performed for tumor resection. Unexpectedly, histopathological examination revealed a germ cell tumor with diffuse infiltration of the dura and bone, characterized by a high Ki-67 labeling index and immunoreactivity for placental alkaline phosphatase. Subsequent systemic evaluation identified a mass in the anterior mediastinum, leading to a final diagnosis of intracranial metastasis from a primary mediastinal seminoma. The patient was referred for systemic chemotherapy and achieved a complete response. Germ cell tumorscannot be excluded in young male patients, even when lesions arise on the convexity and exhibit imaging features typical of meningiomas. Accordingly, germ cell tumors should be included in the differential diagnosis, and tumor marker evaluation and nuclear medicine studies should be proactively performed.

## Introduction

Intracranial germ cell tumors (GCTs) account for <0.5-1% of all central nervous system tumors, and involvement of the cerebral convexity has been only rarely reported. Intracranial germinomas predominantly arise in midline structures, such as the pineal and suprasellar regions [1,2]. Ectopic or metastatic germinomas involving the cerebral convexity without midline involvement are extremely rare [3-5]. GCTs located in the cerebral convexity or frontal lobe are particularly uncommon and often pose substantial diagnostic challenges [6-8]. Due to their location and radiological features, these tumors can closely mimic other pathologies, especially meningiomas [3,6,9]. Because meningiomas are the most common tumors of the cerebral convexity, GCTs are seldom considered in the differential diagnosis. Although a biopsy followed by chemotherapy represents the standard approach when GCTs are suspected, surgical resection may be warranted in patients with large tumor volume and significant mass effect. Herein, we present a rare case of a frontoparietal lobe convexity germinoma, which was ultimately identified as a metastasis from a primary mediastinal seminoma, presenting with dural invasion and skull destruction that closely mimicked a meningioma.

## Case presentation

An 18-year-old man presented to his primary care physician with headache and nausea without any preceding warning signs. Physical examination revealed no obvious findings suggestive of increased intracranial pressure, such as papilledema, and local neurological findings were negative. Non-contrast head computed tomography (CT) revealed an intracranial space-occupying lesion, and he was subsequently referred to our department. The patient had a moderate intellectual disability but was able to perform activities of daily living independently and communicate effectively. At 18 months of age, he had been evaluated for delayed ambulation; however, no abnormalities were identified at that time.

**Figure 1:**
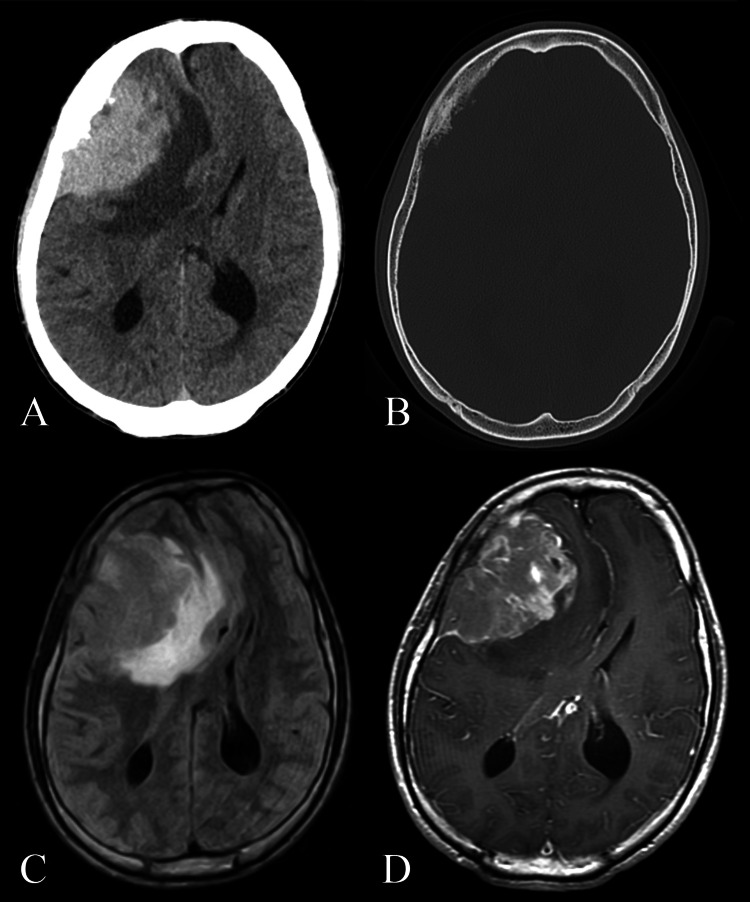
Preoperative images (A) Initial non-contrast head CT scan. (B) Bone window CT image. (C) FLAIR MRI. (D) Gadolinium-enhanced T1-weighted MRI. CT: computed tomography; MRI: magnetic resonance imaging; FLAIR: fluid-attenuated inversion recovery

A CT scan obtained at the referring facility demonstrated a tumor-like lesion in the right frontoparietal region, appearing to originate from the extracranial tissue and causing significant compression of the underlying brain parenchyma. Focal thickening of the adjacent skull bone was also observed at the site of tumor attachment. Upon presentation to our hospital, the patient continued to complain of headache and nausea. Subsequent imaging confirmed tumor-induced compression and edema of the brain parenchyma, resulting in a midline shift. Magnetic resonance imaging (MRI) revealed a lesion that appeared hypointense on T1-weighted images and hyperintense on T2-weighted images. Gadolinium-enhanced MRI demonstrated a mild heterogeneous enhancement of the lesion. Preoperative images are shown in Figure 1.

Based on these imaging findings, a tentative diagnosis of frontoparietal convexity meningioma was made, consistent with radiological features commonly observed in meningioma mimics [9], and craniotomy was considered indicated. The patient was started on isosorbide to reduce intracranial pressure and was scheduled for tumor resection approximately two weeks later. At a follow-up visit five days later, the patient developed a severe headache and nausea, necessitating hospitalization for further management of intracranial pressure. Upon admission, hyperosmotic glycerol-fructose infusion was administered three times daily. His clinical symptoms subsequently improved, and follow-up imaging demonstrated a mild reduction in cerebral edema.

**Figure 2:**
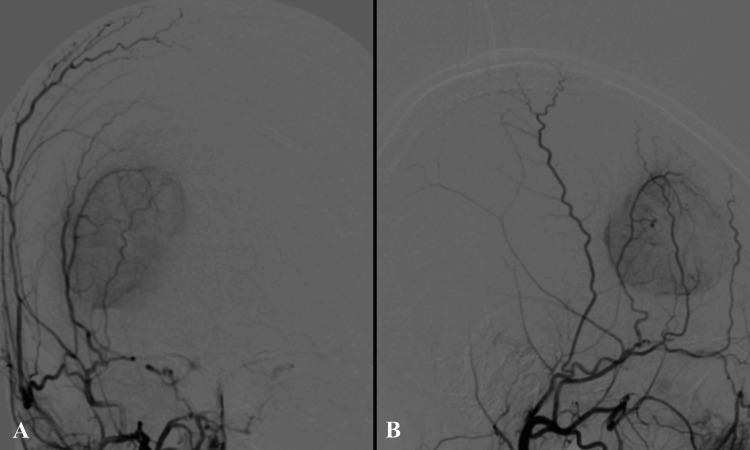
Cerebral angiography (A) Frontal and (B) lateral cerebral angiographic views demonstrating tumor staining supplied by branches of the middle meningeal artery. The feeding arteries originated from the dural attachment and penetrated the tumor interior. Selective preoperative embolization of these branches was performed using granular embolic material.

Craniotomy for tumor resection was performed as scheduled. To minimize intraoperative bleeding, endovascular tumor embolization was performed the day prior to surgery. Angiography identified the anterior convexity branch of the right middle meningeal artery and an anterior branch arising from the pterional segment as tumor-feeding vessels, with confirmed tumor enhancement (Figure 2). The anterior convexity branch was embolized using gelatin microspheres, whereas the anterior branch was embolized with platinum coils due to observed blood flow to the orbital tissues. Post-embolization angiography demonstrated near-complete resolution of tumor enhancement.

**Figure 3:**
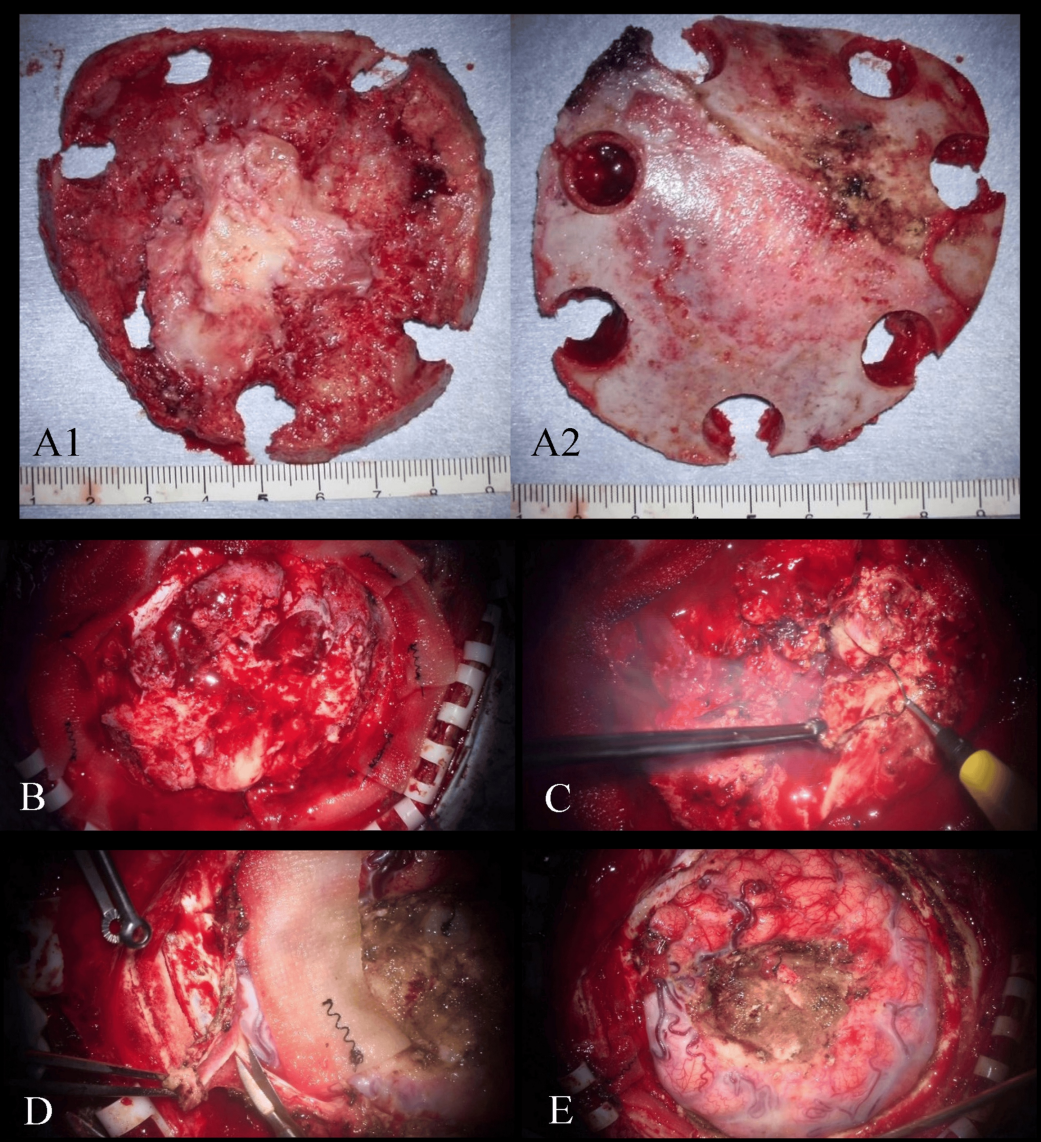
Intraoperative findings (A1, A2) The inner surface of the elevated bone flap. (B) The exposed tumor surface immediately following craniotomy. (C) Internal decompression and resection of the tumor. (D) Excision of the surrounding dura mater. (E) The surgical field following complete tumor removal and dural reconstruction.

Under general anesthesia, a right frontotemporal craniotomy was performed. Upon elevation of the bone flap, the tumor was found to be tightly adherent to both the dura mater and the skull, necessitating the transection of the tumor during bone flap elevation. The inner surface of the bone flap appeared hyperemic, raising suspicion of tumor invasion. A radial dural incision exposed the tumor. Although oozing from the tumor surface was observed, no active arterial bleeding was evident, indicating that the preoperative embolization had been effective. The tumor exhibited an elastic consistency and was initially not readily aspirated. However, internal tumor debulking and decompression using a monopolar ring electrode and an ultrasonic aspirator (CUSA®; Integra LifeSciences, Princeton, NJ, USA) successfully fragmented the tissue, allowing for efficient removal. The residual tumor capsule was meticulously dissected from the brain surface, and the involved dura was resected with an adequate safety margin. Notably, tumor-like tissue extended along the inner dural layer, closely resembling the "dural tail sign" typically observed in meningiomas. Following complete tumor removal, the compressed brain parenchyma re-expanded satisfactorily. Cellulose cotton was applied to prevent postoperative bleeding, and the resulting dural defect was repaired using an artificial dural inlay (DuraGen®; Integra LifeSciences) reinforced with a watertight onlay of an autologous galeal patch. Intraoperative findings are shown in Figure 3.

Postoperative CT revealed a small subcutaneous hematoma that gradually increased in size, raising concern for wound dehiscence. On postoperative day 7, the hematoma was surgically evacuated under general anesthesia. The wound subsequently healed without complications, and the sutures were removed as scheduled.

**Figure 4:**
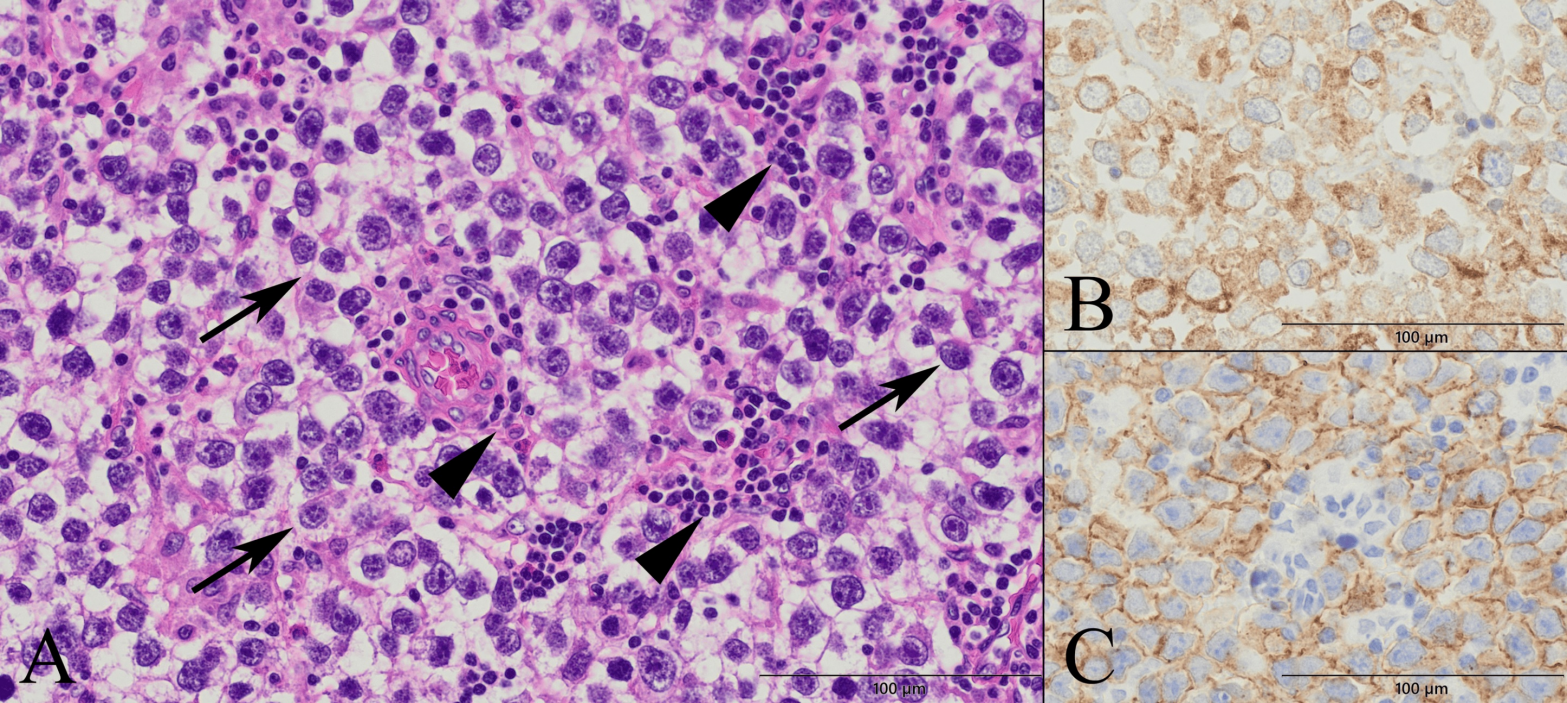
Histopathological findings of the resected intracranial tumor (A) Hematoxylin and eosin (H&E) staining demonstrates the characteristic "two-cell pattern" consisting of a solid proliferation of large atypical cells (arrows) with clear cytoplasm, enlarged vesicular nuclei, and prominent nucleoli, intermingled with small lymphocytes (arrowheads) predominantly in the perivascular areas. These features are strongly consistent with germinoma. Immunohistochemical staining reveals positivity for (B) placental alkaline phosphatase (PLAP) and (C) c-Kit in the tumor cells.

Histopathological examination of the tumor specimen demonstrated a solid proliferation of large atypical cells resembling germ cells. These cells possessed enlarged vesicular nuclei, prominent nucleoli, and clear cytoplasm. An abundant vascular network was interposed between the cell nests, accompanied by small lymphocytic infiltration predominantly in the perivascular areas. This characteristic "two-cell pattern" strongly suggested a germinoma (Figure 4). Serum tumor markers, including alkaline phosphatase and β-human chorionic gonadotropin (β-hCG), were negative. Immunohistochemical analysis showed that the atypical tumor cells were positive for placental alkaline phosphatase (PLAP) and c-Kit, but negative for epithelial membrane antigen (EMA), cytokeratin 5/6 (CK5/6), α-fetoprotein (AFP), and β-hCG. Furthermore, the intermingled small lymphocytes were positive for CD3 and CD20, whereas the tumor cells were negative for these markers. Although STAT6 was also positive, the absence of spindle cells and patternless architecture, combined with strong positivity for specific germ cell markers (PLAP and c-Kit), definitively excluded a solitary fibrous tumor (SFT). The Ki-67 labeling index was approximately 70%, indicating high proliferative activity. These comprehensive pathological findings were highly consistent with a pure seminoma. Diffuse infiltrative tumor cell involvement was observed in both the resected dura mater and bone flap specimens. A plain CT of the trunk revealed the absence of abnormalities. The patient was discharged three weeks postoperatively without neurological deficits and was referred to another institution for further evaluation and chemotherapy.

**Figure 5:**
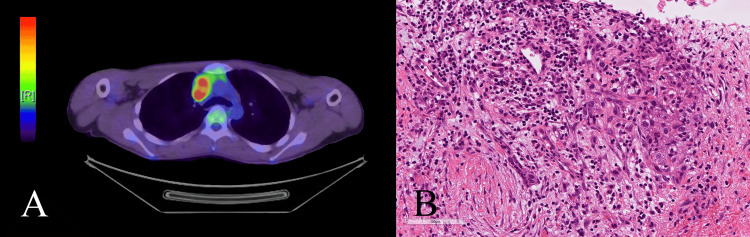
Imaging and histopathological findings of the anterior mediastinal lesion (A) FDG-PET/CT image showing a 43-mm mass with significant FDG uptake in the anterior mediastinum. (B) H&E staining of the CT-guided biopsy specimen from the mediastinal mass, demonstrating the characteristic two-cell pattern. FDG-PET/CT: fluorodeoxyglucose positron emission tomography-computed tomography; H&E: hematoxylin and eosin; CT: computed tomography

At the receiving institution, whole-body fluorodeoxyglucose positron emission tomography-computed tomography (FDG-PET/CT) revealed a 43-mm mass lesion with significant FDG uptake in the anterior mediastinum. No abnormal FDG uptake was observed at other sites. A subsequent CT-guided needle biopsy of the mediastinal lesion was performed. Histopathological examination of the biopsy specimen using hematoxylin and eosin (H&E) staining demonstrated the characteristic "two-cell pattern" comprising tumor cells and lymphocytes, identical to the findings of the intracranial lesion. These clinical and pathological features were consistent with a pure seminoma, solidly supporting the final diagnosis of an intracranial metastasis originating from a primary mediastinal germ cell tumor (Figure 5). The patient subsequently received four cycles of bleomycin, etoposide, and cisplatin chemotherapy, followed by four cycles of paclitaxel, ifosfamide, and cisplatin chemotherapy, administered with curative intent. Although a transient thymic rebound was observed during follow-up, a complete response was achieved. Following the completion of treatment, the patient was transferred to another institution for ongoing care. Recent updates indicate that he remains clinically stable with no evidence of tumor recurrence.

## Discussion

This case represents an exceptionally rare instance of an intracranial metastasis from a primary mediastinal seminoma in an 18-year-old man. Intracranial GCTs account for <0.5-1% of all central nervous system tumors, with most occurring between 10 and 19 years of age and exhibiting a marked male predominance [1,2]. Although the pineal and suprasellar regions are the most frequent sites of involvement, the cerebral convexity is rarely affected [3,4]. Because meningiomas are the most common tumors arising from the cerebral convexity, metastatic or ectopic GCTs are rarely considered in the differential diagnosis for extra-axial lesions in this region [10]. However, in young men, both primary and metastatic GCTs should be considered regardless of tumor location, as several patients with frontal or frontoparietal GCTs presenting with atypical symptoms have been reported [5-8].

When evaluating convexity extra-axial tumors in young patients, a broad differential diagnosis must be considered alongside typical meningiomas [10]. Solitary fibrous tumors (formerly hemangiopericytomas) often present with dural attachment, prominent flow voids, and heterogeneous contrast enhancement, closely resembling the imaging profile of the current case [11]. Granulomatous diseases, such as Langerhans cell histiocytosis or Rosai-Dorfman disease, can also manifest as dural-based masses with associated osteolytic skull destruction [12]. Furthermore, intracranial manifestations of hematological malignancies, such as dural lymphomas or leukemic infiltrates (chloromas), as well as metastatic lesions from extracranial primary malignancies (e.g., Ewing sarcoma or mediastinal GCTs), can present as extra-axial masses with a "dural tail" sign. Recognizing these varied imaging features, particularly heterogeneous enhancement, the presence of prominent flow voids, and lytic bone destruction rather than typical hyperostosis, is critical for differentiating these aggressive entities from benign meningiomas in the young demographic [10].

In the present patient, the initial radiological impression strongly suggested a convexity meningioma due to apparent dural attachment and skull invasion. The "dural tail sign," although typically associated with meningiomas, can occasionally be observed in other tumor types, contributing to diagnostic confusion [9]. Furthermore, the preoperative CT in this case demonstrated hyperostosis-like thickening rather than obvious bone destruction, which further complicated the differential diagnosis [6,13]. A retrospective review of the imaging, however, revealed features atypical of meningiomas, such as heterogeneous contrast enhancement with multiple flow voids [13]. Similar cases of metastatic or ectopic frontoparietal lobe convexity GCTs with dural invasion mimicking meningiomas have been described in the literature [6]. These imaging characteristics, combined with the patient's young age, should prompt a higher index of suspicion for non-meningiomatous lesions.

Accurate diagnosis is essential because pure seminomas/germinomas are highly radiosensitive and potentially curable with chemoradiotherapy, which helps avoid unnecessary radical surgical resection [14]. The definitive diagnosis of a GCT necessitates a comprehensive assessment combining imaging findings, serum tumor markers, and nuclear medicine studies [15]. Tumor markers provide valuable diagnostic and prognostic insights, and even modest elevations can support the diagnosis and serve as a tool for evaluating treatment response [15]. Additionally, positron emission tomography-CT aids in tumor localization and the detection of metastatic disease.

A major limitation of our clinical management in this case was the omission of preoperative serum tumor markers and systemic imaging. Because the clinical and radiological presentations so strongly mimicked a typical convexity meningioma, an extra-axial metastatic germ cell tumor was not initially suspected. Had these comprehensive evaluations been performed preoperatively, an accurate diagnosis might have been reached earlier, potentially altering the initial surgical strategy.

Although biopsy followed by chemotherapy is generally the standard approach for suspected GCTs, surgical tumor resection is appropriate in cases involving large tumor volumes or pronounced mass effects, as seen in this patient [14]. Preoperative vascular embolization was performed to minimize intraoperative bleeding. While not routinely indicated for convexity lesions with readily accessible feeding vessels, embolization was highly effective given the tumor's hypervascularity, further highlighting its resemblance to a meningioma. Following surgery, the patient experienced improvement in neurological symptoms, and complete remission was achieved with adjuvant chemotherapy, demonstrating that craniotomy did not adversely affect the overall clinical outcome.

## Conclusions

Convexity GCTs are extremely rare and may closely mimic meningiomas, including features such as dural invasion and skull destruction. However, this diagnosis should not be excluded in young men, even when lesions exhibit classic radiological characteristics of convexity meningiomas. Retrospectively, atypical imaging findings, particularly heterogeneous enhancement, should prompt consideration of GCTs. Serum tumor markers and nuclear medicine studies should be proactively performed in young patients to reduce the risk of misdiagnosis. The accumulation of additional cases is essential to refine diagnostic criteria and establish optimal therapeutic strategies.
